# THE ROLE OF AGE-FRIENDLY HEALTH CARE IN OLDER ADULTS WITH CANCER

**DOI:** 10.1093/geroni/igad104.0651

**Published:** 2023-12-21

**Authors:** Irene Darmadi Blackberry, Stacey Rich, Oliver Hodge, Tshepo Rasekaba, Nicole Webb, Christopher Steer

**Affiliations:** La Trobe University, Wodonga, Victoria, Australia; La Trobe University, John Richards Centre, Wodonga, Victoria, Australia; University Of NSW, Wodonga, Victoria, Australia; La Trobe University, Wodonga, Victoria, Australia; Albury Wodonga Health, Albury, New South Wales, Australia; Border Medical Oncology and Haematology, Albury, New South Wales, Australia

## Abstract

It is estimated that 51% of cancer incidence worldwide occurs among adults aged 65 years and older. The care of older adults diagnosed with cancer is complex due to an interplay of biopsychosocial factors, including comorbidities, geriatric syndromes, functional status, nutritional health and socioeconomic characteristics. Working within an age-friendly model of care is important in ensuring the complexity of ageing is taken into consideration, while understanding what matters most to the patient undergoing cancer treatment. Age-friendly models of care such as the 4Ms framework (Mentation, Mobility, Medications, and (what) Matters) are gaining support across the globe, but little is known of the use of age-friendly models of care in cancer care. This scoping review aimed to understand the current state of age-friendly care in cancer care and to highlight how age-friendly models can be implemented in cancer care to improve the experience of treatment for older adults.

